# Early universal use of oral progesterone for prevention of preterm births in singleton pregnancy (SINPRO study): protocol of a multicenter, randomized, double-blind, placebo-controlled trial

**DOI:** 10.1186/s13063-020-4067-z

**Published:** 2020-01-30

**Authors:** Ka Wang Cheung, Mimi Tin Yan Seto, Ernest Hung Yu Ng

**Affiliations:** Department of Obstetrics and Gynaecology, Queen Mary Hospital, University of Hong Kong, 6/F, Professorial Block, Queen Mary Hospital, 102 Pokfulam Road, Hong Kong, Hong Kong Special Administration Region China

**Keywords:** Preterm birth, Progesterone, Cervical length, Universal use

## Abstract

**Background:**

Preterm birth accounts for 75% of perinatal deaths and more than 50% of long-term neurological disabilities. For a singleton pregnancy, progesterone treatment is effective in prevention of preterm birth in women with an asymptomatic short cervix or a history of preterm birth. However, a large proportion of preterm births still is not currently preventable. The aim of this study is to determine whether early universal use of oral progesterone before 14 + 0 weeks of gestation can prevent preterm birth better than universal screening of cervical length at 18 + 0 to 23 + 6 weeks of gestation, followed by progesterone treatment in those with a short cervix in singleton pregnancy.

**Methods:**

This is a multicenter, randomized, double-blind, placebo-controlled trial registered with ClinicalTrials.gov on 12 February 2018. Eligible consecutive pregnant women with singleton gestation attending antenatal outpatient clinics will be recruited after receiving counseling and signing the written consent form. Transvaginal cervical length measurement will be performed at recruitment (before 14 + 0 weeks of gestation) and between 18 + 0 and 23 + 6 weeks of gestation. After randomization, women will be randomly assigned to either the treatment group (oral dydrogesterone 10 mg three times daily) or the placebo group, and medication will be started before 14 + 0 weeks of gestation. Assigned groups will be unblinded if the cervical length is ≤ 25 mm between 18 + 0 and 23 + 6 weeks of gestation, and the management option for short cervix will be discussed (oral progesterone, vaginal progesterone, or cervical cerclage). The primary outcome is preterm birth before 37 + 0 weeks of gestation.

**Discussion:**

Progesterone is used extensively in part of the in vitro fertilization program as luteal phase support, and it is not associated with teratogenicity. Universal progesterone supplementation may be a better approach to prevent preterm birth. This large, multicenter, randomized, double-blind, placebo-controlled trial will provide the best evidence, leading to the best strategy for the prevention of preterm birth.

**Trial registration:**

ClinicalTrials.gov, NCT03428685. Registered on 12 February 2018.

## Background and rationale

Preterm birth (PTB), defined as birth before 37 completed gestational weeks, is a major challenge to perinatal health. It accounts for 75% of perinatal deaths and more than 50% of long-term neurological disabilities [1, 2], and it is the second most common cause of death in children under the age of 5 years [3]. Neonates born preterm are at risk of respiratory distress syndrome, chronic lung disease, retinopathy of prematurity, necrotizing enterocolitis, intraventricular hemorrhage, and sepsis in the short term, as well as cerebral palsy, motor and sensory impairment, learning difficulties, and increased risk of chronic disease in long run [4]. The estimated societal cost of PTB is $26 billion annually in the United States alone. The cost of PTB is inversely related to gestational age at birth. Costs stand at over US$100,000, US$40,000–US$100,000, US$10,000–US$30,000, and below US$4500 for babies born at extreme prematurity (< 28 weeks of gestation), early prematurity (28–31 weeks of gestation), moderate prematurity (32–34 weeks of gestation), and late prematurity (35–36 weeks of gestation), respectively [5]. On the basis of evaluation of data from 184 countries, the estimated global mean PTB rate is approximately 11.1%, ranging from 5% to 18% in different countries [6].

For singleton pregnancy, prevention of PTB is based on identification of risk factors in obstetrical history, biochemical markers, and short cervix. Both history of PTB and asymptomatic short cervix at the second trimester are good predictors of PTB [7–10]. Transvaginal cervical length measurement (TVCL) at approximately 24 weeks of gestation can predict PTB before 35 weeks using a cutoff for cervical length of ≤ 25 mm with sensitivity of 37.3%, specificity of 92.2%, and positive and negative predictive values of 17.8% and 97.0%, respectively [7].

Progesterone, by its maintenance of uterine quiescence through anti-inflammatory effects, has been shown to decrease the risk of PTB in women with the aforementioned risk factors [11, 12]. In women with prior PTB, the weekly use of 250 mg of 17α-hydroxyprogesterone caproate intramuscularly could reduce the PTB rate before 37 weeks of gestation from 54.9% to 36.3% (relative risk [RR], 0.66; 95% confidence interval [CI], 0.54–0.81) [11]. In women with asymptomatic short cervix (TVCL measurement of 10–20 mm) at the second trimester, 90 mg of vaginal progesterone could effectively reduce PTB before 33 weeks from 16.1% to 8.9% (RR, 0.55; 95% CI, 0.33–0.92) [13]. Universal cervical length screening followed by treatment with vaginal progesterone has been shown to be the most cost-effective strategy for preventing PTB [14, 15]. These findings were confirmed in a meta-analysis [16].

Nevertheless, only a minority of women may benefit from progesterone treatment if it is started at the second trimester. This is because only approximately 10% of spontaneous early PTB cases were associated with a history of prior PTB [17] and approximately 5% of women had a cervical length of approximately 20 mm at 22 + 0–24 + 6 weeks of gestation [7]. Therefore, the remaining women who may have PTB may not have any identifiable risk factor. Even if physicians are able to detect these risk factors and offer progesterone treatment at approximately 16–24 weeks, 36.3% of women with a history of PTB and 30.2% of women with an asymptomatic short cervix still give birth before 37 weeks [11, 13]. There is still a large proportion of PTB, which is currently not preventable, and the current approach to preventing PTB is far from ideal. One possible hypothesis is that the initiation of progesterone treatment would be too late for its effect to take place. Early progesterone treatment may maintain uterine quiescence and prevent shortening of the cervix from early gestation [18].

### Objectives and hypothesis

#### Primary objective

The aim of this multicenter, randomized, double-blind, placebo-controlled trial is to determine whether early universal use of oral progesterone before 14 + 0 weeks of gestation can prevent PTB in singleton gestations better than universal screening of cervical length at 18 + 0 to 23 + 6 weeks of gestation, followed by progesterone treatment in those with a short cervix in a singleton pregnancy. The hypothesis is that early universal progesterone treatment from before 14 + 0 weeks could decrease the risk of PTB further than progesterone treatment in women with an asymptomatic short cervix detected by universal cervical length screening.

## Methods

### Trial design

**Fig. 1:**
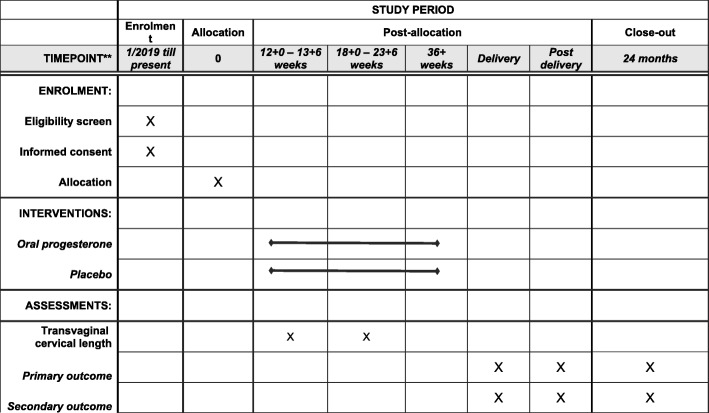
Schedule of enrollment, intervention, and assessment

SINPRO (Early Use of Oral Progesterone in All Women for Prevention of Preterm Delivery in Singleton Pregnancy) is a multicenter, randomized, double-blind, placebo-controlled trial that will be carried out in six public maternity units in Hong Kong: Kwong Wah Hospital, Pamela Youde Nethersole Eastern Hospital, Princess Margaret Hospital, Queen Elizabeth Hospital, Queen Mary Hospital, and United Christian Hospital. The steering committee consists of the principal investigator and co-investigators from other centers. The data management team is composed of a group of maternal and fetal subspecialists who will meet every 6 months to oversee the progress and safety of the study, which is independent from the funder and competing interests. These recruiting hospitals serve approximately 55% of the births in Hong Kong. Queen Mary Hospital is affiliated with the University of Hong Kong. The study has been approved by the Institutional Review Board of the University of Hong Kong/Hospital Authority Hong Kong West Cluster (UW 17-308) and has been registered with ClinicalTrials.gov (identifier NCT03428685). The clinical trial protocol follows the Standard Protocol Items: Recommendations for Interventional Trials (SPIRIT) 2013 checklist format (Additional file 1). If any change to the protocol is required, the funder and the ethics committee will be notified. The principal investigator will then inform the participating centers and update the information in the clinical trial registry. Deviations from the protocol will be fully documented using a breach report form. Figure 1 summarizes the trial design, and the details are described below. The study started in January 2019 and had recruited 310 subjects by 13/1/2020.

### Eligibility criteria

Women attending the antenatal service will be informed of this study. Research assistants will visit the participating hospitals regularly to ensure adequate subject enrollment. Women aged ≥ 18 years who have a confirmed viable singleton intrauterine pregnancy before 14 + 0 weeks of gestation will be recruited. They will have a dating scan before 14 + 0 weeks of gestation to confirm no dating problem, and the estimated date of confinement will be based on the ultrasound scan findings. Women will be excluded if they meet any of the following criteria:

1. Miscarriage
2. Ectopic pregnancy
3. Twin pregnancy with miscarriage of one twin
4. Heavy vaginal bleeding requiring surgical intervention
5. Severe abdominal pain requiring surgical intervention
6. Presence of fever
7. History of adverse reaction to progesterone
8. History of breast or genital tract malignancy
9. History of suspected thromboembolic disease
10. Congenital uterine anomaly
11. Unwillingness or inability to comply with study procedures
12. Known paternal or maternal abnormal karyotype

Women who are willing to join the study will sign the consent form prior to any procedures being done. The investigators will sign the consent form. The consented women can withdraw their consent at any time without affecting the standard medical care they receive. On the consent form, participants will be asked for permission for the research team to share relevant data with people from the hospitals taking part in the research or from regulatory authorities, where relevant. This trial involves collecting biological specimens for storage.

### Randomization and masking

The randomization process will be done in a 1:1 ratio by a research nurse not involved in the women’s clinical care, using a computer program. The result of each randomized participant will be put in a sequentially numbered, opaque, sealed envelope. One envelope will be opened if a woman agrees to join the study. Women will be randomly assigned at the time of recruitment to one of the two groups on the day of first TVCL measurement before 14 + 0 weeks of gestation:

1. *Treatment group*: dydrogesterone 10 mg three times daily (Abbott Biologicals B.V., Olst, The Netherlands)
2. *Placebo group*: placebo identical to the active drug in external appearance but without an active ingredient

Participating women, attending clinicians, study investigators, and study coordinators are blinded to the group assigned.

### Procedure

Ultrasonographers must attend the “Cervical Assessment” online lecture and obtain the certificate of competence from the Fetal Medicine Foundation website. TVCL will be measured using a standard protocol [19]. Women will be asked to empty the urinary bladder before the TVCL measurement. A transvaginal ultrasound probe will be inserted into the vagina and directed into the anterior fornix. A sagittal view of the cervical canal will be obtained, and excessive pressure on the cervix will be avoided. Cervical length will be defined as the linear measurement of the cervical canal between the V-shaped internal and external os, surrounded by the hypoechoic endocervical mucosa. The shortest distance between the three measurements will be recorded. If uterine contraction is observed in the lower segment close to the internal os, ultrasound will be repeated after 5–10 min when the contraction ceases. After the baseline cervical length assessment, women will be instructed to take the oral progesterone or placebo accordingly, three times per day, from 12 + 0 to 14 + 0 weeks to 36+ weeks of gestation.

A second TVCL assessment will be performed between 18 + 0 weeks and 23 + 6 weeks of gestation. On the basis of available evidence of progesterone treatment in women with asymptomatic short cervix [12], women in the assigned group will be unblinded if the TVCL measurement is ≤ 25 mm. The women receiving placebo will be given the option of oral progesterone (10 mg three times per day) or vaginal progesterone 200 mg daily (Endometrin, Ferring Pharmaceuticals Inc., Parsippany, NJ, USA; or Utrogestan, Besins Healthcare, Monaco) until 36+ weeks of gestation (or cervical cerclage if the women has a history of preterm delivery or second-trimester miscarriage) [20]. For women in the progesterone group whose cervical length is ≤ 25 mm at 18 + 0 to 23 + 6 weeks of gestation, options will be offered of continuation of oral progesterone (10 mg three times per day), vaginal progesterone 200 mg daily (Endometrin or Utrogestan), or cervical cerclage if the women have a history of PTB.

Progesterone level will be obtained at the time of recruitment, between 18 + 0 and 23 + 6 weeks, and between 28 + 0 and 31 + 6 weeks of gestation. Serum will be drawn at the time of recruitment for further analysis. Participating women will be asked to return the empty drug packaging at follow-up to check for compliance and to remind them to complete the follow-up. Adverse effects from the medication will also be noted. Satisfactory compliance will be defined as taking at least 80% of prescribed medication. Treatment will be stopped if there is rupture of membranes or established preterm labor (cervical dilation ≥ 3 cm). Women will be followed up at the antenatal clinic as scheduled and managed according to departmental protocol. During and after the study period, subjects will be managed by the investigators, who have ample experience in looking after obstetric patients and managing them throughout their pregnancy. There is no compensation for trial participation.

### Outcomes

The primary outcome is the rate of PTB **(**defined as birth before 37 + 0 gestational weeks). Outcomes will also be evaluated for subjects who discontinue or deviate from the protocol. Other secondary outcomes and their definitions are as follows:

#### Obstetric outcomes

- Spontaneous PTB before 37 + 0 weeks
- PTB before 32 + 0 weeks
- PTB before 28 + 0 weeks
- Preterm prelabor rupture of membrane (rupture of membrane before 37 + 0 weeks, confirmed clinically by [a] oozing of liquor from the external cervical os, [b] pooling of liquor at the posterior fornix, or [c] decreased liquor on ultrasound with history suggestive of leaking)
- Spontaneous miscarriage before 20 + 0 weeks
- Stillbirth: fetus dies in utero after 24 + 0 weeks
- Cervical length at 18 + 0 to 23 + 6 weeks
- Gestational age at birth
- Mode of delivery

#### Maternal outcomes

- Gestational hypertension: development of new-onset hypertension (blood pressure persistently ≥ 140/90 mmHg on two occasions at least 4 h apart during pregnancy after 20 weeks of gestation, labor, or the puerperium in previously normotensive nonproteinuric women
- Preeclampsia: gestational hypertension with proteinuria
- Gestational proteinuria: spot urine for initial estimation of total protein excretion of 300 mg or more/24 h
- Gestational diabetes: using a 75-g 2-h oral glucose tolerance test, any of the fasting glucose ≥ 5.1 mmol/L, 1-h plasma glucose ≥ 10 mmol/L, or 2-h plasma glucose ≥ 8.5 mmol/L
- Antepartum hemorrhage: any vaginal bleeding during pregnancy from 24 weeks to term
- Nausea
- Vomiting
- Serum progesterone level at recruitment, between 18 + 0 and 23 + 6 weeks and between 28 + 0 and 31 + 6 weeks

#### Neonatal outcomes

- Birth weight
- Apgar score
- Small for gestational age (<10th percentile for birth weight)
- Head circumference: measure at the occipital frontal level
- Congenital anomaly
- Neonatal intensive care unit admission
- Intraventricular hemorrhage, diagnosed by ultrasound scan
  - ▪ Grade 1: germinal matrix hemorrhage only or germinal matrix hemorrhage plus intraventricular hemorrhage < 10% of ventricular area
  - ▪ Grade 2: intraventricular hemorrhage, 10–50% of ventricular area
  - ▪ Grade 3: intraventricular hemorrhage involving > 50% of ventricular area; lateral ventricles are usually distended
  - ▪ Grade 4: parenchymal bleed in any location
- Severe intraventricular hemorrhage (grade 3 or 4 periventricular-intraventricular hemorrhage)
- Cystic periventricular leukomalacia
- Necrotizing enterocolitis above stage 1, defined by modified Bell staging criteria as stage IIA or above
- Bronchopulmonary dysplasia
- Respiratory distress syndrome (radiological evidence of respiratory distress syndrome)
- Severe retinopathy of prematurity (stage 3 ROP retinopathy of prematurity or higher)
- Proven sepsis
- In-hospital death
- Length of stay
- Hemodynamically significant patent ductus arteriosus requiring ligation

### Statistics

#### Statistical tests

##### Analysis plan

All statistical analyses will be carried out using SAS version 9.2 or above (SAS Institute, Cary, NC, USA) and/or R version 3.2.0 or above (R Foundation for Statistical Computing, Vienna, Austria) or IBM SPSS Statistics software (IBM, Armonk, NY, USA).

The primary analysis will be evaluated by Fisher’s exact test to compare the difference of PTB rates between the progesterone and control groups. The analysis will primarily be carried out according to intention-to-treat and per-protocol principles. The intention-to-treat analysis will include any randomized subjects with nonmissing primary outcome data. The per-protocol analysis will include all randomized subjects in the intention-to-treat analysis who have sufficient treatment compliance (≥ 80%) and no major protocol deviations.

Nominal data will be described by frequencies and percentages, and correlations will be analyzed using the chi-square test; continuous data will be expressed as mean ± standard deviation or median (range) and analyzed using Student’s *t* test or the Mann-Whitney *U* test, depending on the normality of the data. Logistic regression analysis will be applied to evaluate the association between PTB and the effect of early universal use of progesterone, history of previous PTB, cervical length measurement at 18 + 0 to 23 + 6 weeks of gestation, serum progesterone level, and other risk factors, if appropriate. The interaction effect will be evaluated to assess the impact of the history of previous PTB or cervical length on treatment effect. A *P* value < 0.05 will be considered as statistically significant. Interim analysis will be done when the primary outcome is available for 850 subjects (~ 50% of the intended recruitment), and the trial will be stopped if there is a significant difference in the PTB rate between the two groups.

The research assistants will be responsible for data entry. Hard copies will be locked in a cabinet during the course of the study, and the electronic copy will be encrypted/password-protected. The data collected during the course of the research will be kept strictly confidential and will be accessible only by members of the trial team. Anonymized trial data can be shared with other researchers to enable international meta-analyses.

#### Sample size estimation

The PTB rate of singleton pregnancy at 37 weeks of gestation in Hong Kong is approximately 6.5% [21]. Among the local population, 4.6% had cervical length < 25 mm [22]. The use of universal screening of cervical length followed by treatment with vaginal progesterone in those with a short cervix would further bring down the rate to 6% [23]. Treatment with progesterone is anticipated to further reduce this proportion by an absolute difference of 3%. A sample size of 814 per group is required to achieve power of 80% power at the 5% significant level based on Fisher’s exact test. Assuming an attrition rate of 5%, a total of 1714 subjects (857 subjects per group) will be recruited.

## Discussion

Progesterone is used extensively as part of the in vitro fertilization program as luteal phase support [24], in addition to use as treatment for prevention of PTB [12]. It is not associated with teratogenicity and is usually well tolerated [25, 26]. Universal progesterone supplementation may be a feasible approach to preventing PTB. The compliance with use of vaginal progesterone (defined as ≥ 80% use) for prevention of PTB was suboptimal (66%) [27]. Because oral progesterone has also been associated with PTB reduction [28, 29], we decided to use oral progesterone in the current study. This large-scale, multicenter, randomized controlled study will provide data and inform the best strategy for the prevention of PTB. The results of the trial will be published in a peer-reviewed journal.

## Trial status

The study started in January 2019 and had recruited 310 subjects by 13/1/2020.

Protocol 4.0. Date 3/9/2018.

## Supplementary information

**Additional file 1.** SPIRIT 2013 Checklist: Recommended items to address in a clinical trial protocol and related documents.

## Data Availability

The datasets used and/or analyzed during the current study are available from the corresponding author on reasonable request. The consent form is available from the corresponding author on request.

## Abbreviations

PTB: Preterm birth
TVCL: Transvaginal cervical length

## References

[1] Tucker J, McGuire W (2004). Epidemiology of preterm birth. BMJ.

[2] Goldenberg RL, Culhane JF, Iams JD, Romero R (2008). Epidemiology and causes of preterm birth. Lancet.

[3] Liu L, Johnson HL, Cousens S, Perin J, Scott S, Lawn JE (2012). Global, regional, and national causes of child mortality: an updated systematic analysis for 2010 with time trends since 2000. Lancet.

[4] Mwaniki MK, Atieno M, Lawn JE, Newton CR (2012). Long-term neurodevelopmental outcomes after intrauterine and neonatal insults: a systematic review. Lancet.

[5] Soilly AL, Lejeune C, Quantin C, Bejean S, Gouyon JB (2014). Economic analysis of the costs associated with prematurity from a literature review. Public Health.

[6] Blencowe H, Cousens S, Oestergaard MZ, Chou D, Moller AB, Narwal R (2012). National, regional, and worldwide estimates of preterm birth rates in the year 2010 with time trends since 1990 for selected countries: a systematic analysis and implications. Lancet.

[7] Iams JD, Goldenberg RL, Meis PJ, Mercer BM, Moawad A, Das A, National Institute of Child Health and Human Development Maternal Fetal Medicine Unit Network (1996). The length of the cervix and the risk of spontaneous premature delivery. N Engl J Med.

[8] Spong CY (2007). Prediction and prevention of recurrent spontaneous preterm birth. Obstet Gynecol.

[9] Owen J, Yost N, Berghella V, Thom E, Swain M, Dildy GA (2001). Mid-trimester endovaginal sonography in women at high risk for spontaneous preterm birth. JAMA.

[10] Crane JM, Hutchens D (2008). Transvaginal sonographic measurement of cervical length to predict preterm birth in asymptomatic women at increased risk: a systematic review. Ultrasound Obstet Gynecol.

[11] Meis PJ, Klebanoff M, Thom E, Dombrowski MP, Sibai B, Moawad AH (2003). Prevention of recurrent preterm delivery by 17α-hydroxyprogesterone caproate. N Engl J Med.

[12] Romero R, Conde-Agudelo A, Da Fonseca E, O’Brien JM, Cetingoz E, Creasy GW (2018). Vaginal progesterone for preventing preterm birth and adverse perinatal outcomes in singleton gestations with a short cervix: a meta-analysis of individual patient data. Am J Obstet Gynecol.

[13] Hassan SS, Romero R, Vidyadhari D, Fusey S, Baxter JK, Khandelwal M (2011). Vaginal progesterone reduces the rate of preterm birth in women with a sonographic short cervix: a multicenter, randomized, double-blind, placebo-controlled trial. Ultrasound Obstet Gynecol.

[14] Einerson BD, Grobman WA, Miller ES (2016). Cost-effectiveness of risk-based screening for cervical length to prevent preterm birth. Am J Obstet Gynecol.

[15] Conde-Agudelo A, Romero R (2016). Vaginal progesterone to prevent preterm birth in pregnant women with a sonographic short cervix: clinical and public health implications. Am J Obstet Gynecol.

[16] Dodd JM, Jones L, Flenady V, Cincotta R, Crowther CA. Prenatal administration of progesterone for preventing preterm birth in women considered to be at risk of preterm birth. Cochrane Database Syst Rev. 2013;(7):CD004947. 10.1002/14651858.CD004947.pub3. 10.1002/14651858.CD004947.pub3 PMC11035916 23903965

[17] Bloom SL, Yost NP, McIntire DD, Leveno KJ (2001). Recurrence of preterm birth in singleton and twin pregnancies. Obstet Gynecol.

[18] Facchinetti F, Paganelli S, Comitini G, Dante G, Volpe A (2007). Cervical length changes during preterm cervical ripening: effects of 17-α-hydroxyprogesterone caproate. Am J Obstet Gynecol.

[19] Kagan KO, Sonek J (2015). How to measure cervical length. Ultrasound Obstet Gynecol.

[20] Conde-Agudelo A, Romero R, Da Fonseca E, O’Brien JM, Cetingoz E, Creasy GW (2018). Vaginal progesterone is as effective as cervical cerclage to prevent preterm birth in women with a singleton gestation, previous spontaneous preterm birth, and a short cervix: updated indirect comparison meta-analysis. Am J Obstet Gynecol.

[21] Hui AS, Lao TT, Leung TY, Schaaf JM, Sahota DS (2014). Trends in preterm birth in singleton deliveries in a Hong Kong population. Int J Gynaecol Obstet.

[22] Hui SY, Chor CM, Lau TK, Lao TT, Leung TY (2013). Cerclage pessary for preventing preterm birth in women with a singleton pregnancy and a short cervix at 20 to 24 weeks: a randomized controlled trial. Am J Perinatol.

[23] Romero R, Nicolaides K, Conde-Agudelo A, Tabor A, O’Brien JM, Cetingoz E (2012). Vaginal progesterone in women with an asymptomatic sonographic short cervix in the midtrimester decreases preterm delivery and neonatal morbidity: a systematic review and metaanalysis of individual patient data. Am J Obstet Gynecol.

[24] van der Linden M, Buckingham K, Farquhar C, Kremer JA, Metwally M (2015). Luteal phase support for assisted reproduction cycles. Cochrane Database Syst Rev.

[25] Resseguie LJ, Hick JF, Bruen JA, Noller KL, O’Fallon WM, Kurland LT (1985). Congenital malformations among offspring exposed in utero to progestins, Olmsted County, Minnesota, 1936-1974. Fertil Steril.

[26] Raman-Wilms L, Tseng AL, Wighardt S, Einarson TR, Koren G (1995). Fetal genital effects of first-trimester sex hormone exposure: a meta-analysis. Obstet Gynecol.

[27] Norman JE, Marlow N, Messow CM, Shennan A, Bennett PR, Thornton S (2016). Vaginal progesterone prophylaxis for preterm birth (the OPPTIMUM study): a multicentre, randomised, double-blind trial. Lancet.

[28] Ashoush S, El-Kady O, Al-Hawwary G, Othman A (2017). The value of oral micronized progesterone in the prevention of recurrent spontaneous preterm birth: a randomized controlled trial. Acta Obstet Gynecol Scand.

[29] Boelig RC, Della Corte L, Ashoush S, McKenna D, Saccone G, Rajaram S (2019). Oral progesterone for the prevention of recurrent preterm birth: systematic review and metaanalysis. Am J Obstet Gynecol MFM.

